# Supplementary material for: Synthesis and Properties of Modified Biodegradable Polymers Based on Caprolactone
Source: Polymers (Basel). 2023 Dec 17;15(24):4731. doi: 10.3390/polym15244731 (PMC10748277; doi:10.3390/polym15244731)
Supplement: Supplementary file 1 [file polymers-15-04731-s001.zip › polymers-2718062-supplementary.pdf]

polymers

MDPI

Supplementary Materials

Synthesis and Properties of Modified Biodegradable Polymers Based on Caprolactone

Maria E. Fortună [1], Elena Ungureanu [2,*], Răzvan Rotaru [1], Alexandra Bargan [1], Ovidiu C. Ungureanu [3], Carmen O. Brezuleanu [2] and Valeria Harabagiu [1]

[1] Institute of Macromolecular Chemistry "Petru Poni", 41A Grigore Ghica Voda Alley, 700487 Iasi, Romania; fortuna.maria@icmpp.ro (M.E.F.); rotaru.razvan@icmpp.ro (R.R.); anistor@icmpp.ro (A.B.); hvaleria@icmpp.ro (V.H.)
[2] "Ion Ionescu de la Brad" Iasi University of Life Sciences, 3 Mihail Sadoveanu Alley, 700490 Iasi, Romania; olgutabrez@uaiasi.ro
[3] "Vasile Goldis" Western University of Arad, 94 the Boulevard of the Revolution, 310025 Arad, Romania; ungureanu.ovidiu@uvvg.ro
* Correspondence: eungureanu@uaiasi.ro

S1. Preparation of the aminopropyl-terminated polydimethylsiloxane-poly(ε-caprolactone) copolymers: PDMS-PCL-1 and PDMS-PCL-2

The copolymers were synthesized via the ring-opening polymerization of poly-ε-caprolactone (PCL) in the presence of aminopropyl-terminated polydimethylsiloxane (APDMS) with $Sn(Oct)_2$ as the catalyst, as shown in Scheme 1.

Thus, ε-caprolactone (3 g), aminopropyl terminated polydimethylsiloxane (1.6 g), stannous octoate catalyst (0.023 g), and 10 g of xylene were added to a 50 mL three-neck flask at 120 °C and stirred under nitrogen for 24 h when the copolymers were synthesized. The purification of the copolymers was performed by precipitating the reaction mixture in methanol. The precipitated products obtained were dried in a vacuum drying oven at 30–40 °C.

The aminopropyl-terminated polydimethylsiloxane-poly(ε-caprolactone) copolymers (PDMS-PCL) were obtained.

i

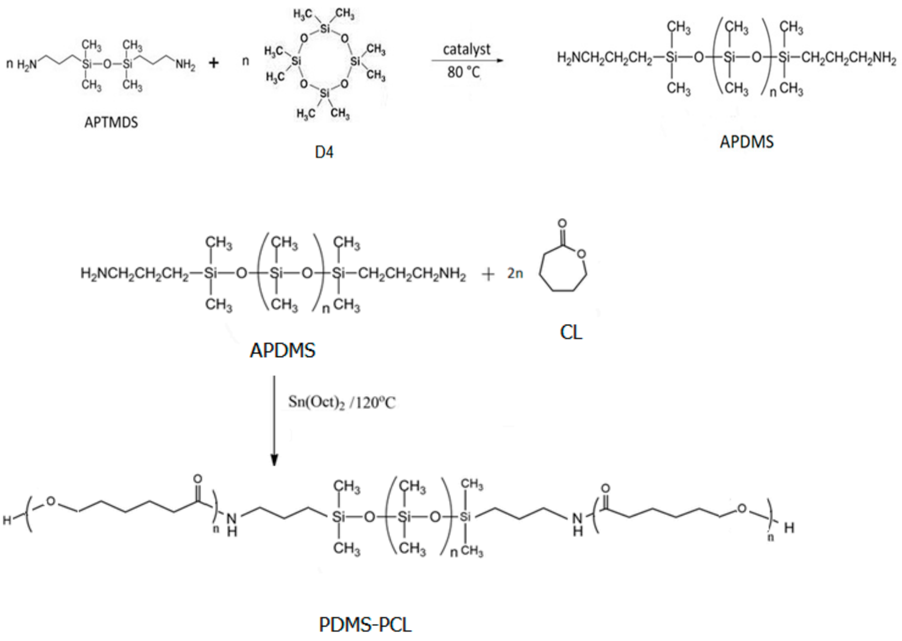

Scheme S1. PDMS-PCL copolymers synthesis.

S2. NMR spectra for PDMS-PCL-1 and PDMS-PCL-2 copolymers

The synthesis of the well-defined copolymer was confirmed by the NMR spectra (Figure S1).

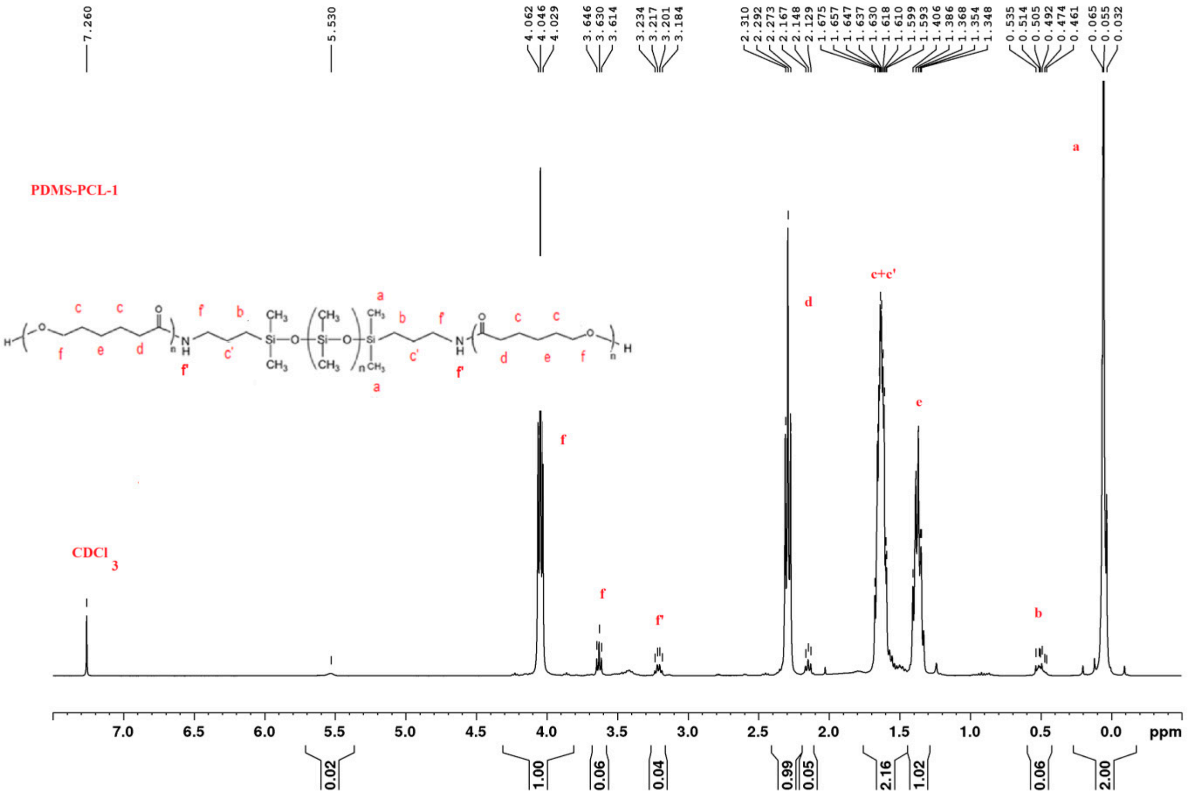

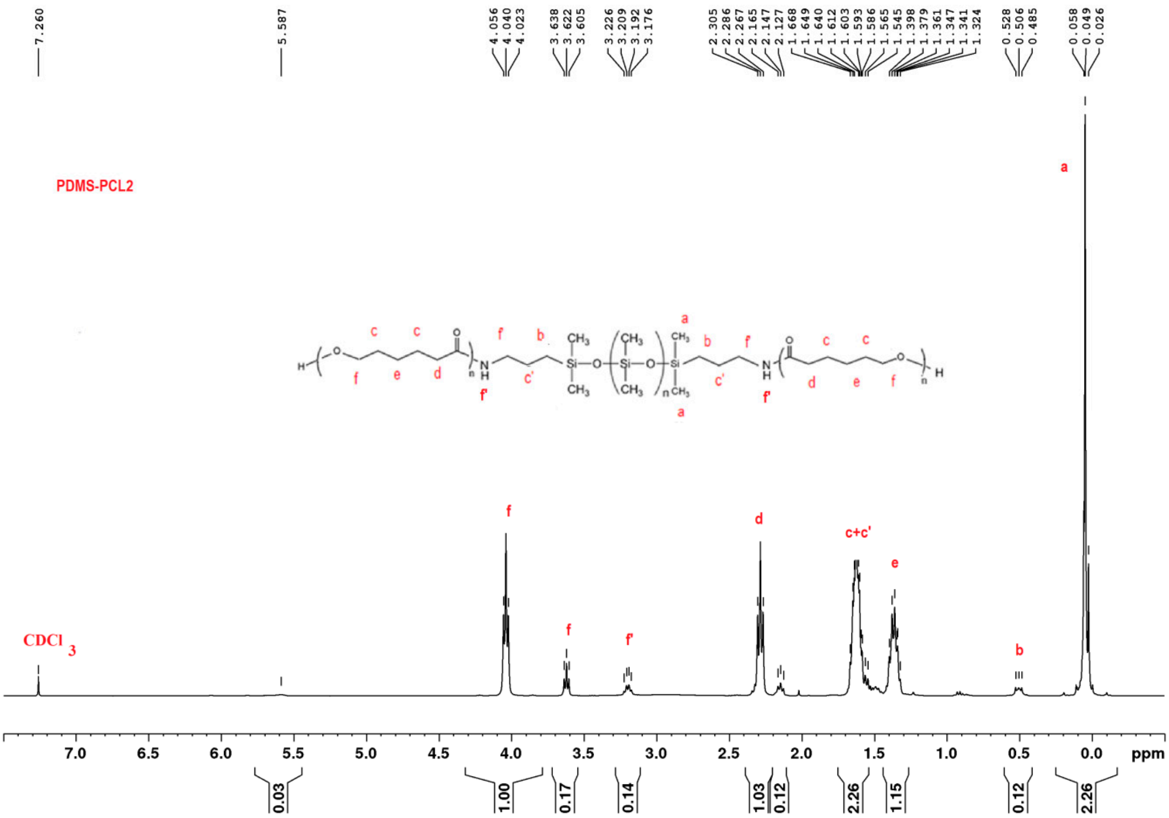

Figure S1. NMR spectra for PDMS-PCL-1 and PDMS-PCL-2 copolymers.

–CH_2–CH_2–CH_2–NH– (1.61 ppm); –CO–CH_2–CH_2–CH_2– (1.40 ppm); –CO–CH_2–CH_2– (1.60 ppm); –CO–CH_2– (2.31 ppm); –Si(CH_3)$_2$– (0.07 ppm); –Si–CH_2–(0.5 ppm); and –CH_2–CH_2–CH_2 (1.47); -NH (3,2); –CH_2–H (3.6 ppm); –CH_2–NH– (4.05 ppm).

Table S1. The total chlorophyll content in the leaves of tomato plants 35 days after planting.

Samples	Chlorophyll content index, 1 CCl					
	Reference	APDMS-1	APDMS-2	PDMS-PCL-1	PDMS-PCL-2	PCL
	10.4	7.2	7.4	8.7	8.8	9.9
	10.4	7.1	7.5	8.6	8.9	9.9
	10.3	7.3	7.4	8.8	8.7	9.9
	10.5	7.2	7.3	8.7	8.8	9.9
